# Contour Porcelain Inlays or Tips with Wire-Loop Attachment

**Published:** 1904-05

**Authors:** W. A. Capon

**Affiliations:** Philadelphia.


### CONTOUR PORCELAIN INLAYS OR TIPS WITH WIRE-LOOP ATTACHMENT.

W. A. CAPON, PHILADELPHIA.

Burnish thin platinum over the surface of the natural tooth to be repaired, and fit a loop or pin of platinum wire, No. 24 gauge, to the interior of the cavity; then hold the matrix and wire in position on tooth, and apply porcelain body mixed with water and gum tragacanth. Carefully draw the combination from the tooth and fuse in furnace. This method avoids soldering. Dr. Capon used the method in the formation of a porcelain tip and corner with a loop and pin attachment for an upper central incisor.—*Cosmos.*

